# Supplementary material for: Treatment of MRSA-infected osteomyelitis using bacterial capturing, magnetically targeted composites with microwave-assisted bacterial killing
Source: Nat Commun. 2020 Sep 7;11:4446. doi: 10.1038/s41467-020-18268-0 (PMC7477539; doi:10.1038/s41467-020-18268-0)
Supplement: Supplementary file 1 — Supplementary Information [file 41467_2020_18268_MOESM1_ESM.pdf]

Supplementary Information

Treatment of MRSA-infected osteomyelitis using bacterial capturing, magnetically targeted composites with microwave-assisted bacterial killing

Qiao *et al*.

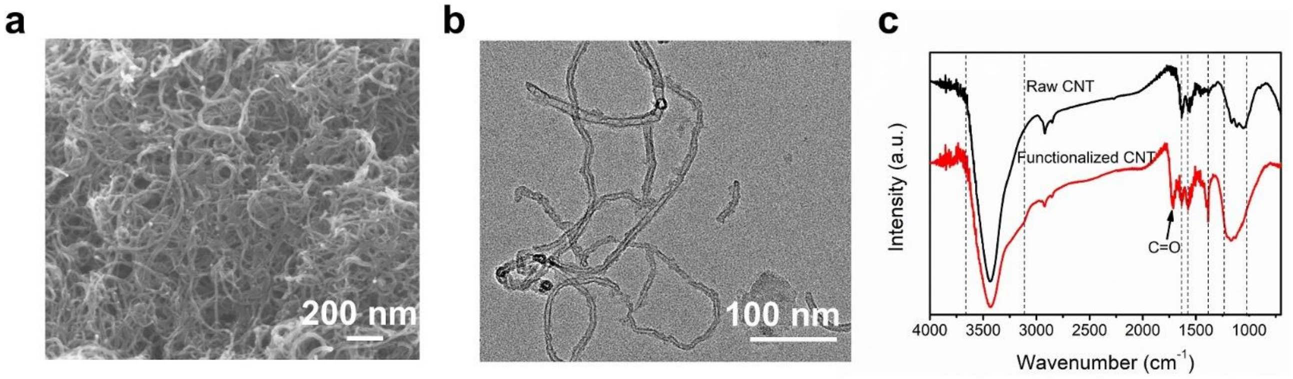

Supplementary Fig. 1 Basic morphology and structure characterization of functionalized CNT. **a,b** SEM image (**a**) and TEM (**b**) image of the functionalized CNT. **c** FTIR spectra of the raw CNT and the functionalized CNT. Unless otherwise specified, all CNT in the text was refers to the functionalized CNT. Source data are provided as a Source Data file.

SEM image showed the network-like morphology of CNT (Supplementary Fig .1 a), and TEM image of CNT showed a hollow nanostructure (Supplementary Fig .1b). As shown in Supplementary Fig. 1c, the peak at 1583 cm^{-1} and 1707 cm^{-1} are attributed to the stretching vibration of C=C stretching in CNTs and C=O in -COOH groups[1], respectively. And the broad peak between 3630 and 3100 cm^{-1} corresponds to the stretching vibration of -OH; and that between 1220 and 1050 $cm^{-1}$is attributed to the vibrations of C-O[2]. These results indicated that the functional CNTs were successfully prepared.

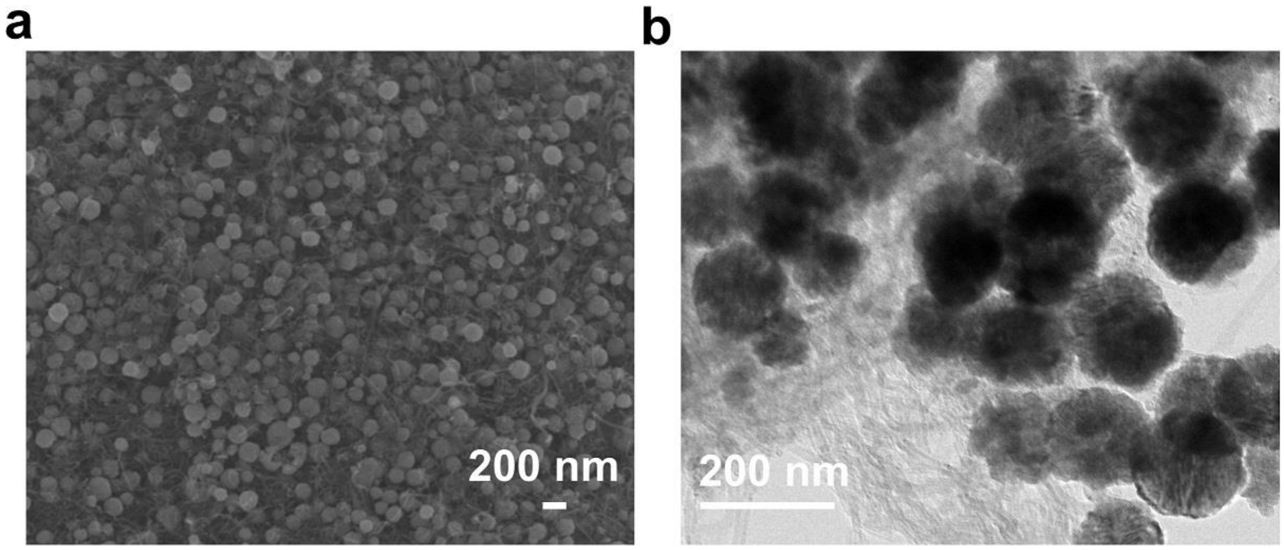

Supplementary Fig. 2 Morphology and structure of Fe_3O_4/CNT. (**a**) SEM image, and (**b**) TEM image.

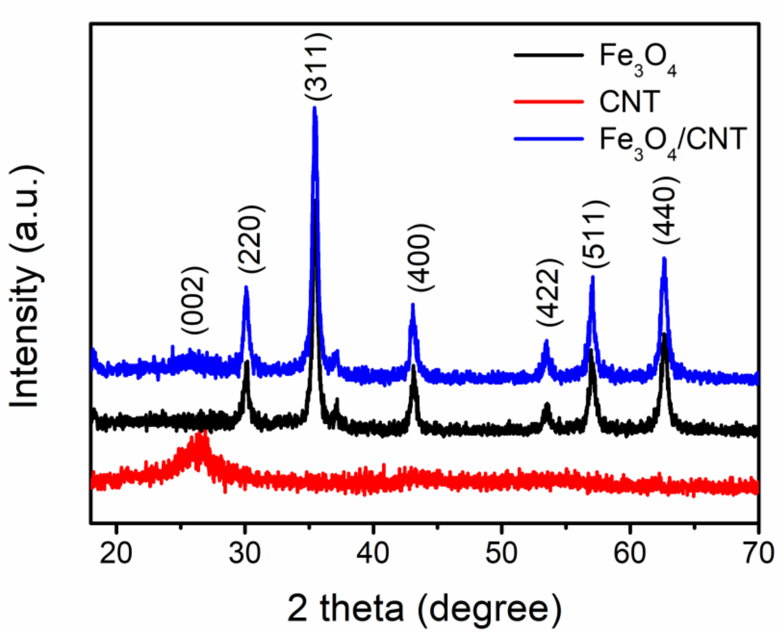

Supplementary Fig. 3 The XRD patterns of Fe_3O_4, CNT, and Fe_3O_4/CNT. Source data are provided as a Source Data file.

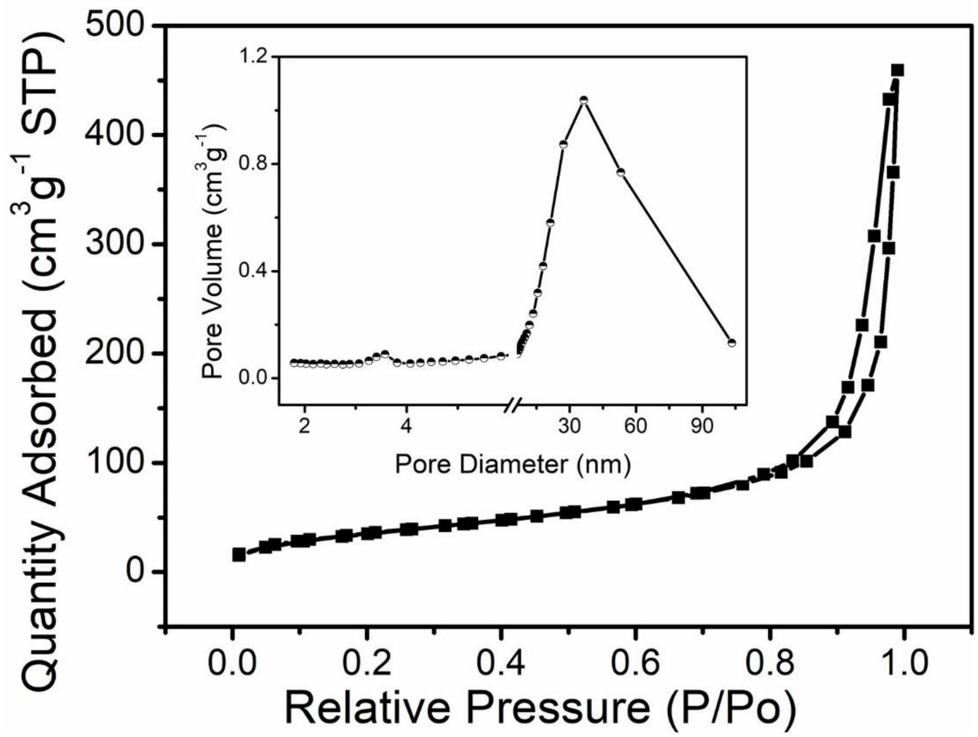

Supplementary Fig. 4 Nitrogen adsorption results for Fe_3O_4/CNT. Inset: The average diameter centered of Fe_3O_4/CNT. Source data are provided as a Source Data file.

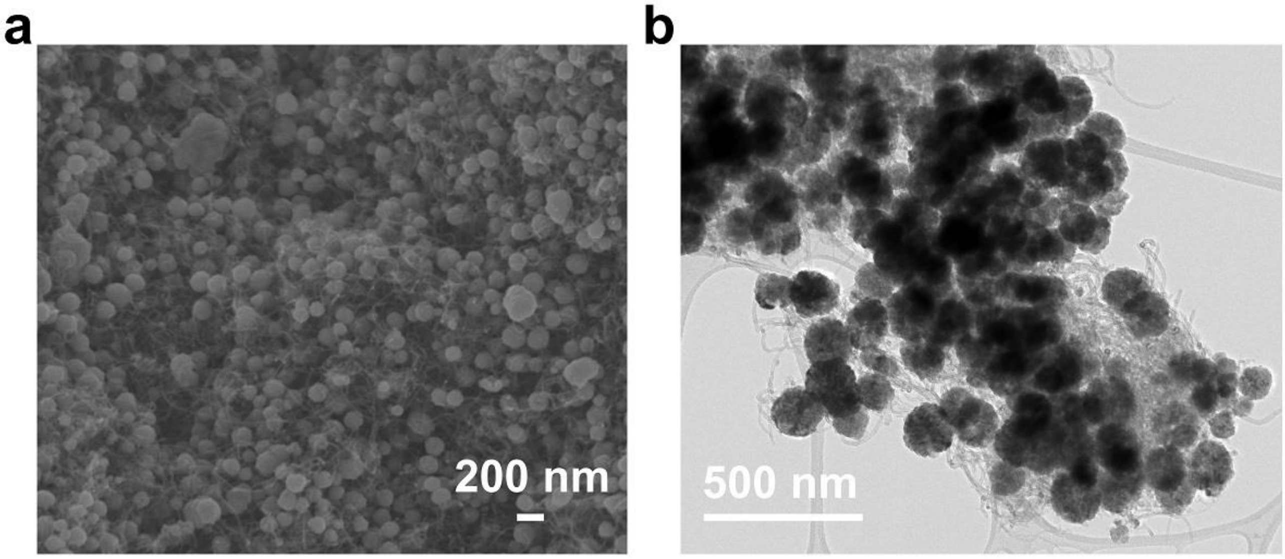

Supplementary Fig. 5 Morphology and structure of Fe_3O_4/CNT/Gent. (**a**) Representative SEM and (**b**) TEM images for Fe_3O_4/CNT/Gent.

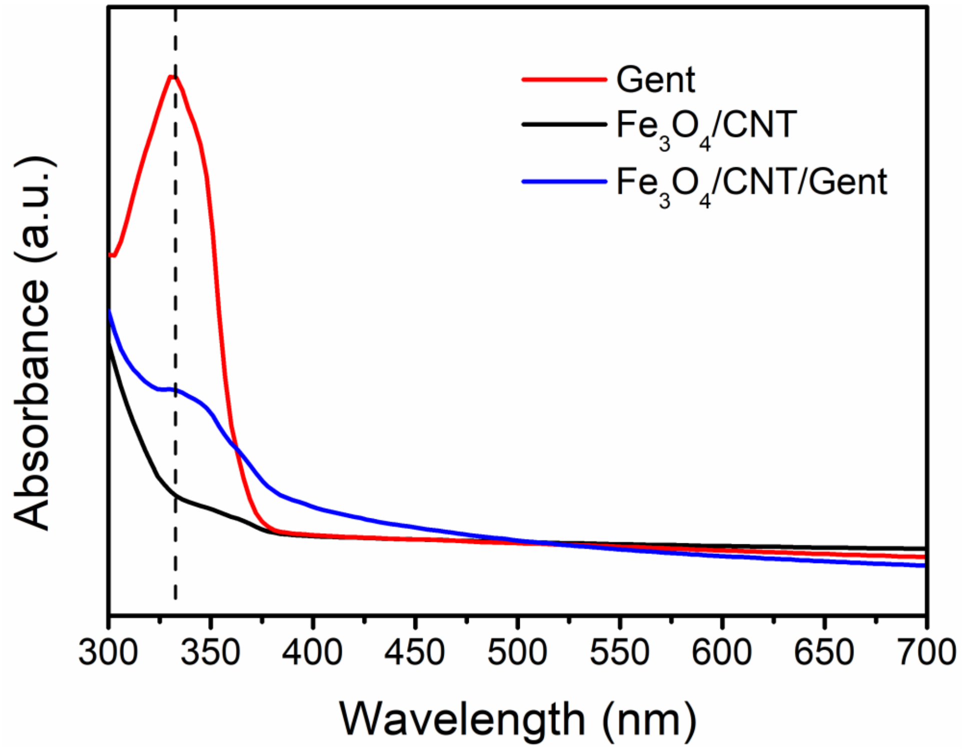

Supplementary Fig. 6. UV-vis absorption spectra of free Gent, Fe_3O_4/CNT, and Fe_3O_4/CNT/Gent. Source data are provided as a Source Data file.

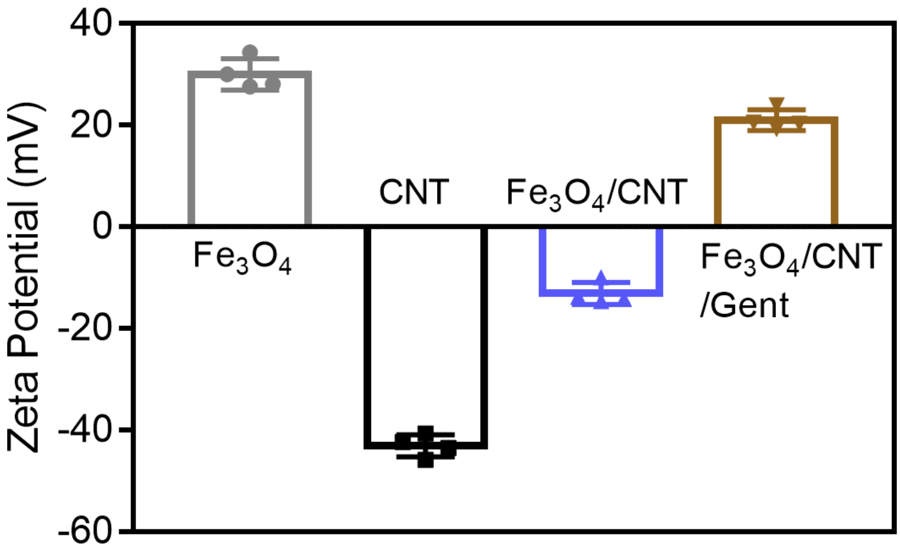

Supplementary Fig. 7 Zeta potential of Fe_3O_4, CNT, Fe_3O_4/CNT, and Fe_3O_4/CNT/Gent. Data are shown as mean ± standard deviations; *n* =4 independent samples. Source data are provided as a Source Data file.

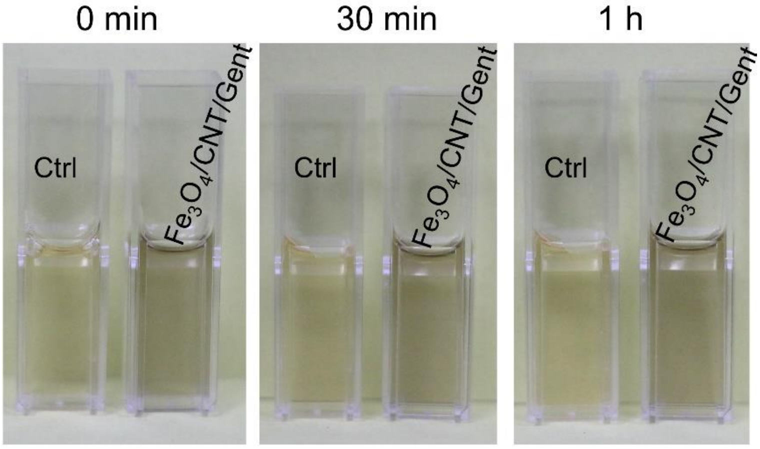

Supplementary Fig. 8 Images of Fe_3O_4/CNT/Gent (100 ppm) in fetal bovine serum setting with different times.

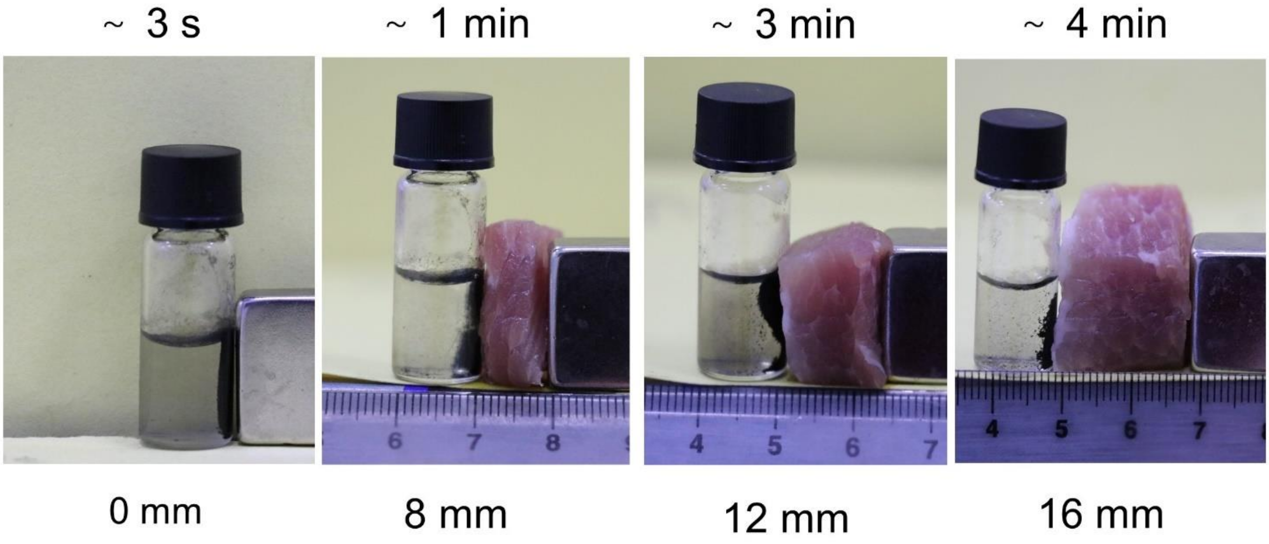

Supplementary Fig. 9 Deep evaluation of the magnetic field attracting Fe_3O_4/CNT/Gent using different thickness of pork. Longer time needed with a magnet behind thicker pork to completely attract Fe_3O_4/CNT/Gent. Neodymium rare earth permanent magnet with grade N38 magnetic energy, around 30 mm × 20 mm × 10 mm in length, width, and height, respectively.

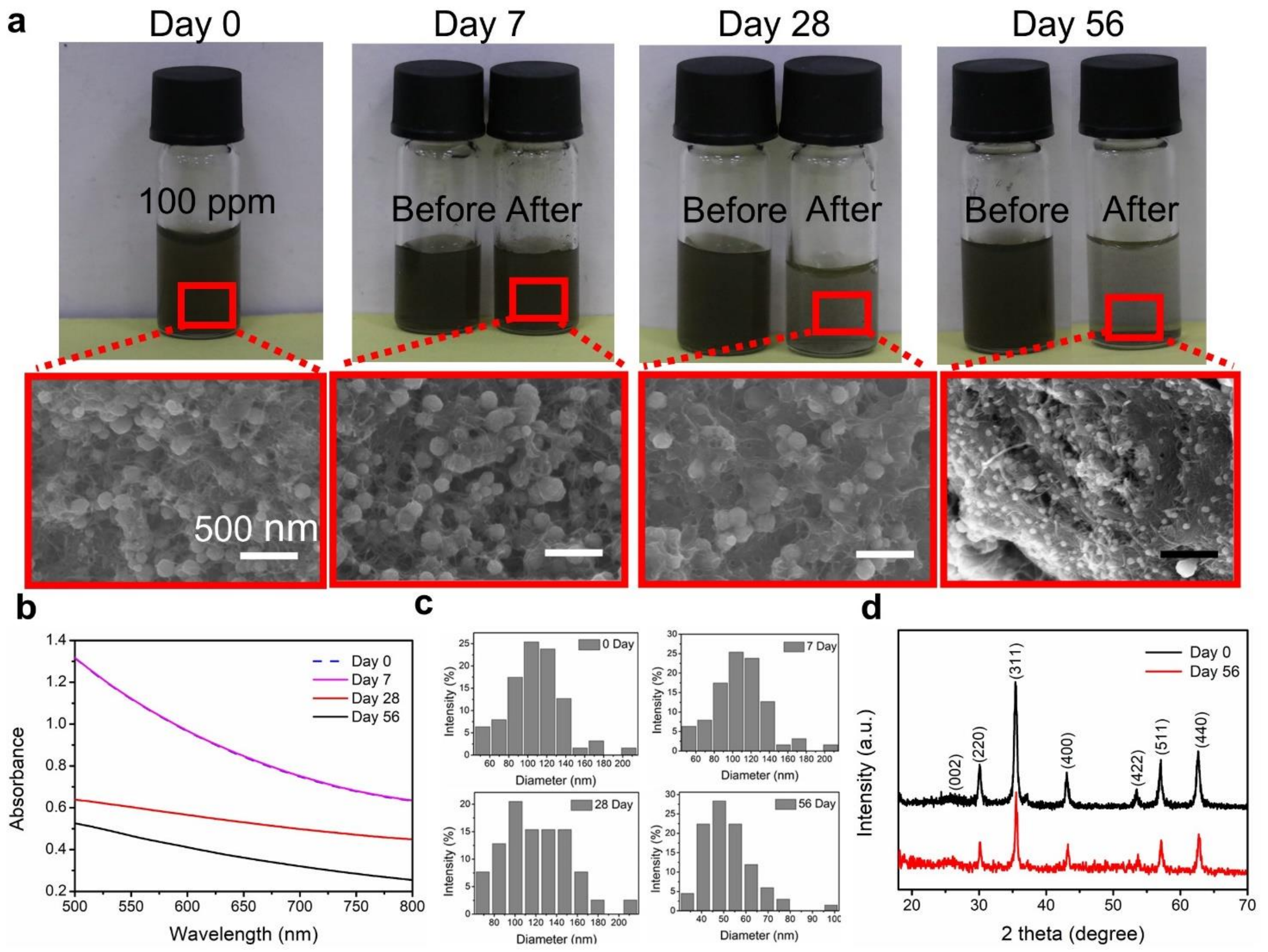

Supplementary Fig. 10 Biodegradation behavior of Fe_3O_4/CNT/Gent. **a,** Digital photograph of Fe_3O_4/CNT/Gent (100 ppm) and corresponding SEM images of 100 ppm Fe_3O_4/CNT/Gent in PBS with shaking at 37°C for 0, 7, 28, and 56 days. **b,** Degradation absorbance curve of Fe_3O_4/CNT/Gent. **c,** Fe_3O_4/CNT/Gent size distribution in PBS with shaking at 37 °C for 0, 7, 28, and 56 days. **d,** The XRD patterns of Fe_3O_4/CNT/Gent in PBS with shaking at 37 °C for 0 and 56 days. Source data are provided as a Source Data file.

The biodegradation behavior of the Fe_3O_4/CNT/Gent was investigated in the phosphate-buffered saline (PBS; pH 7.4) by shaking at 37°C. As shown in Supplementary Fig. 10a, b, compared to the one at Day 0, the Fe_3O_4/CNT/Gent at Day 7 exhibited almost the same absorption curve. Over time, the absorbance of the Fe_3O_4/CNT/Gent gradually reduced; after immersion in PBS for 56 days, it decreased sharply, with obvious color fading and the particle size changed from the original 110 nm to 50 nm (Supplementary Fig. 10c), but crystal structure and composition of Fe_3O_4/CNT/Gent did not show obvious change (Supplementary Fig. 10d), indicating the gradual disintegrability of

Fe_3O_4/CNT/Gent in vitro under our experimental conditions. Especially, previous reports have reported that both Fe_3O_4 and CNT alone can be degraded in vivo, the Fe_3O_4 nanoparticles are processed by cells as part of the physiological iron metabolism[3] and the CNT degraded by neutrophil myeloperoxidase[4], so the degradability of Fe_3O_4/CNT/Gent in vivo is beyond doubt. The disintegrability can favor nanocapturer to be cleared from the body in a reasonable time once it has fulfilled its therapeutic functions in vivo.

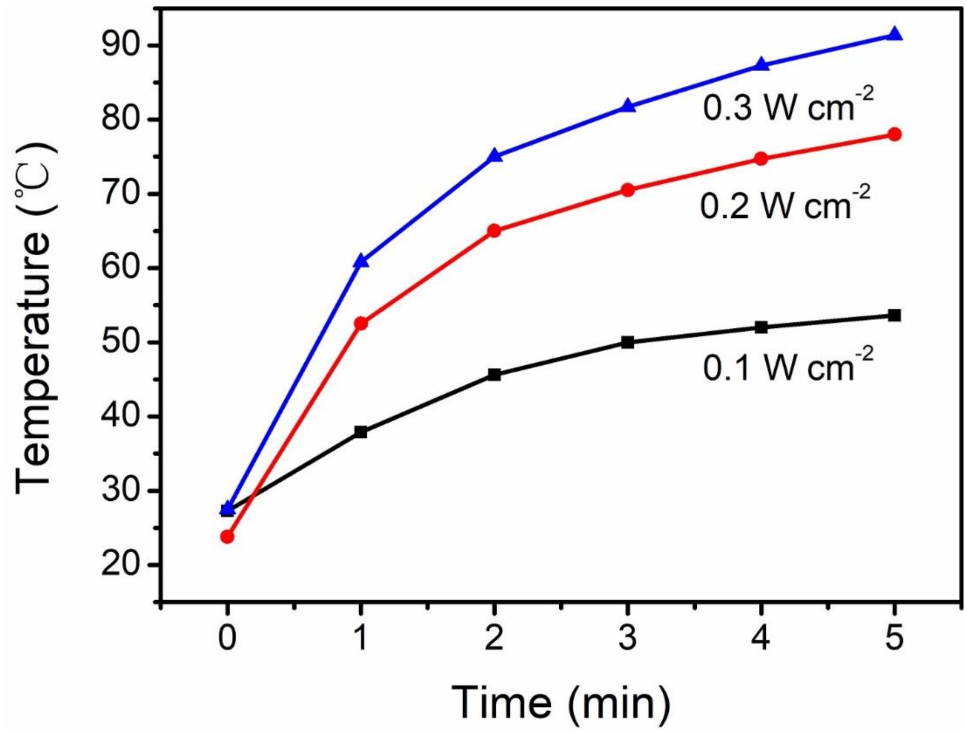

Supplementary Fig. 11 Microwaveocalortic curves of Fe_3O_4/CNT/Gent at different power densities. Microwaveocalortic curves of physiological saline with dispersed Fe_3O_4/CNT/Gent (1mg mL^{-1}) under MV-excited at varied power densities (0.1, 0.2, and 0.3 W cm^{-2}). Source data are provided as a Source Data file.

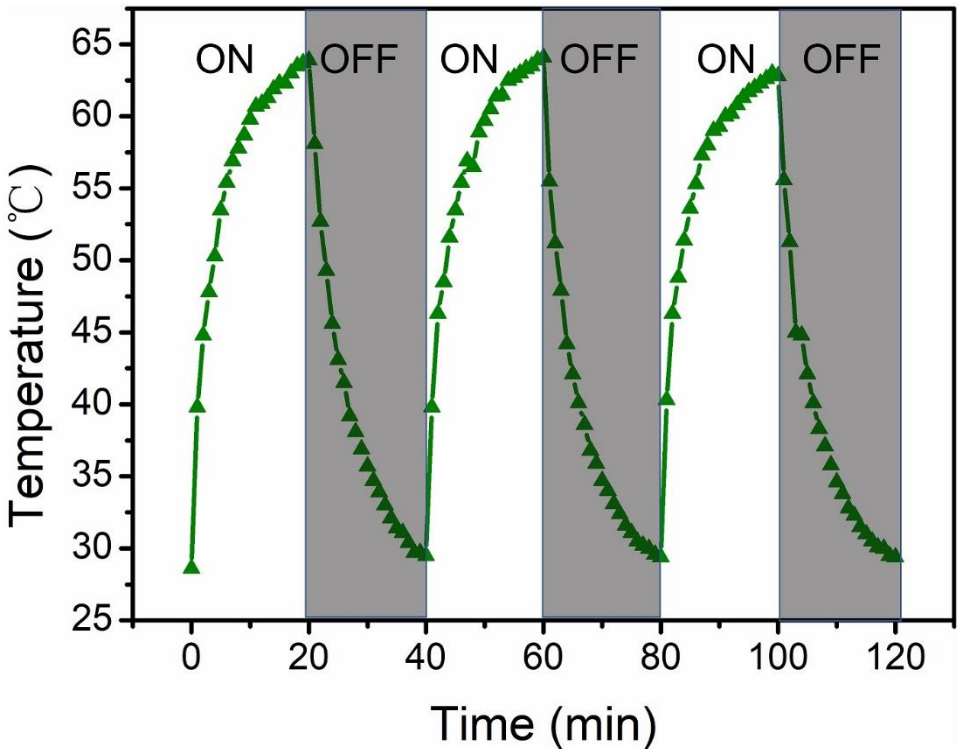

Supplementary Fig. 12 Temperature rising and cooling profiles of the Fe_3O_4/CNT/Gent (1mg mL^{-1}) when the irradiation is on/off under MV (0.1 W cm^{-2}). Source data are provided as a Source Data file.

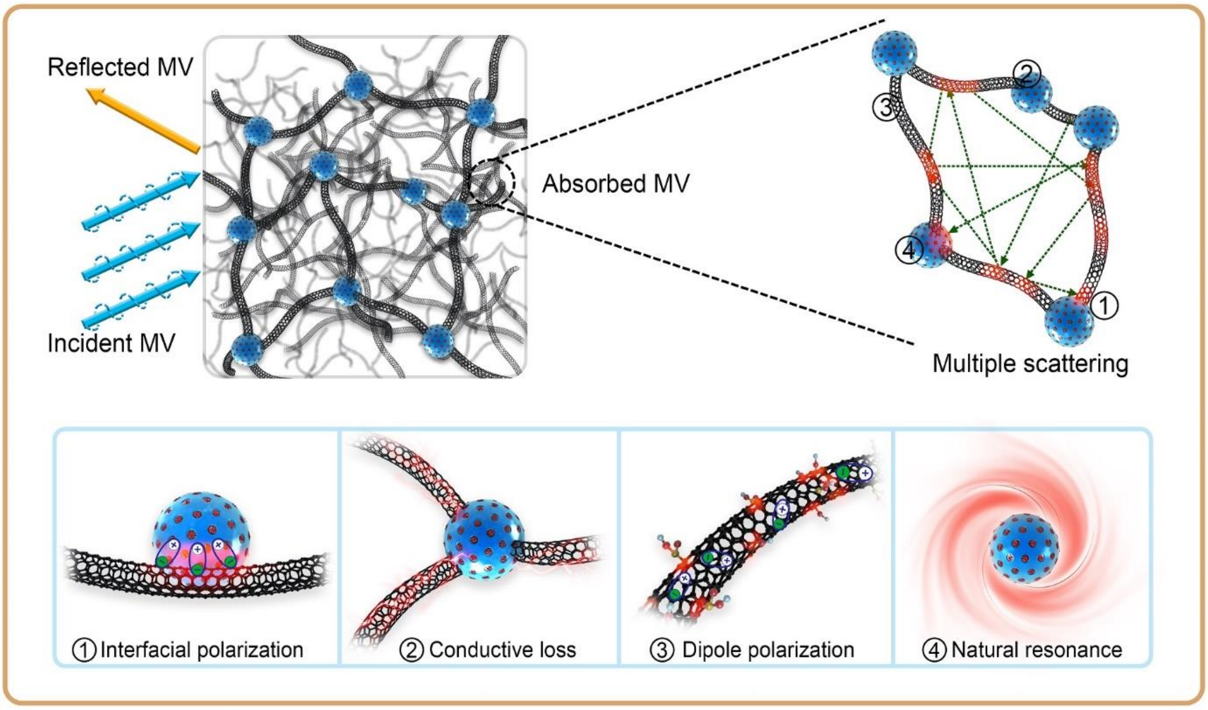

Supplementary Fig. 13 Related microwave absorption mechanism in the Fe_3O_4/CNT/Gent nanocapturer.

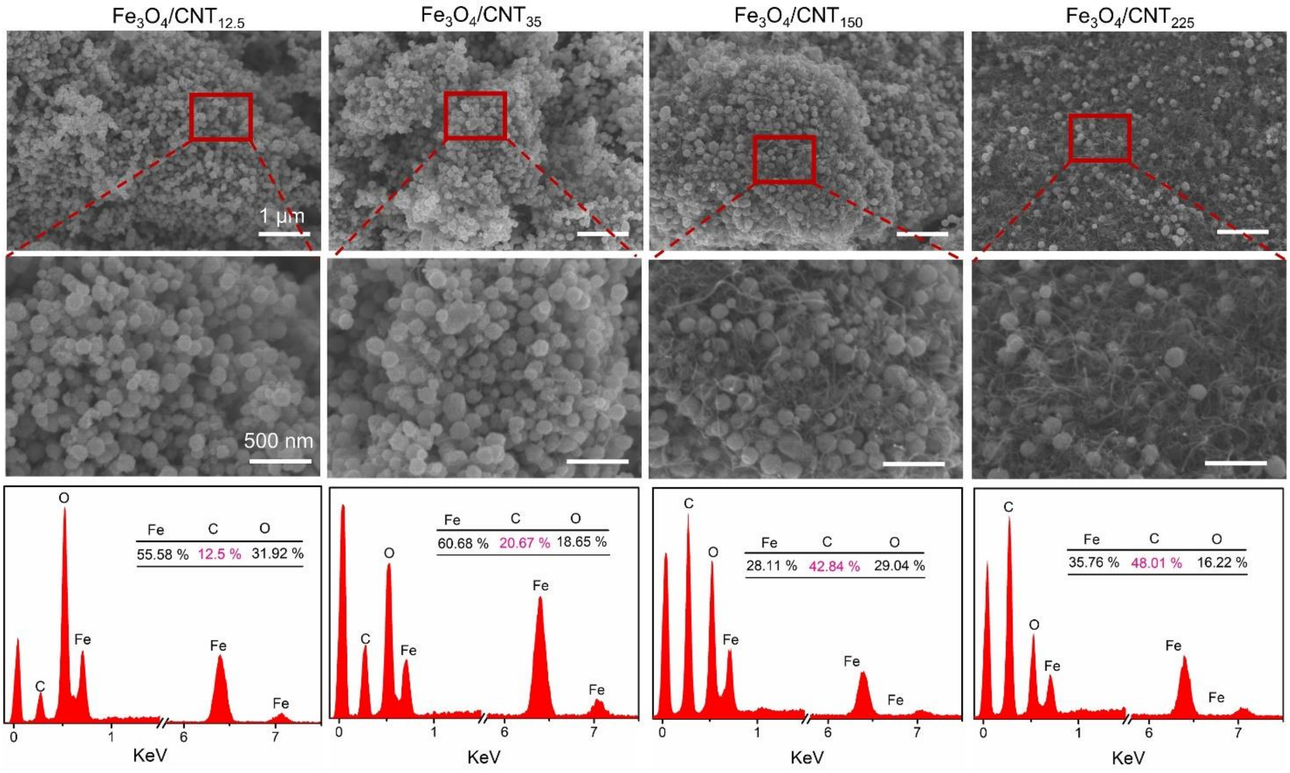

Supplementary Fig. 14 The SEM and EDS images of Fe_3O_4/CNT hybrids with different amounts of CNT. Unless otherwise specified, all Fe_3O_4/CNT in the text was refers to Fe_3O_4/CNT_{150}. Source data are provided as a Source Data file.

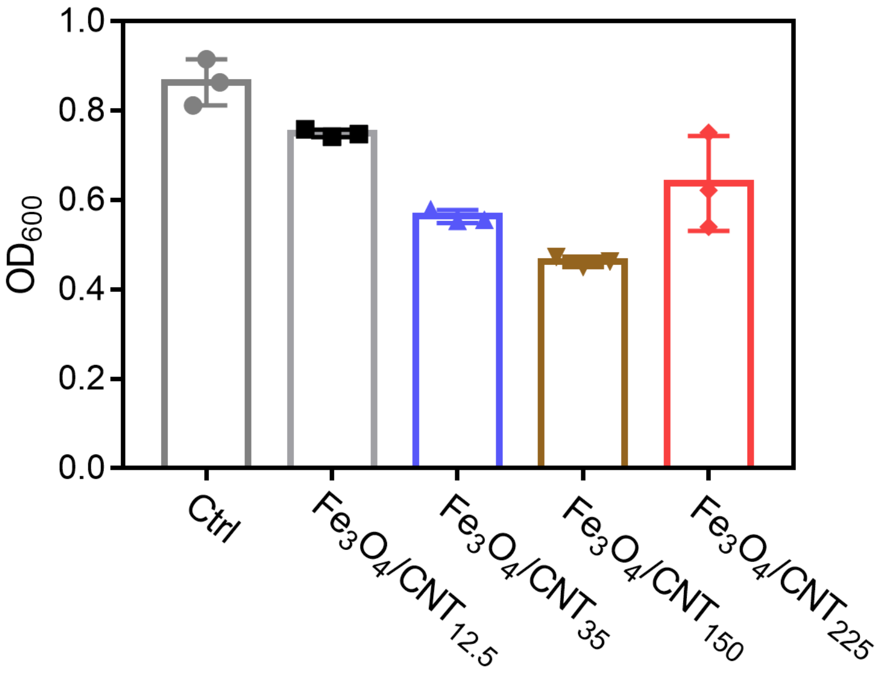

Supplementary Fig. 15 Capturing capacity of Fe_3O_4/CNT hybrids to MRSA. Ctrl: the same volume physiological saline replaced the solution of Fe_3O_4/CNT hybrids (200 ppm). Data are shown as mean ± standard deviations; *n* =3 independent samples. Source data are provided as a Source Data file.

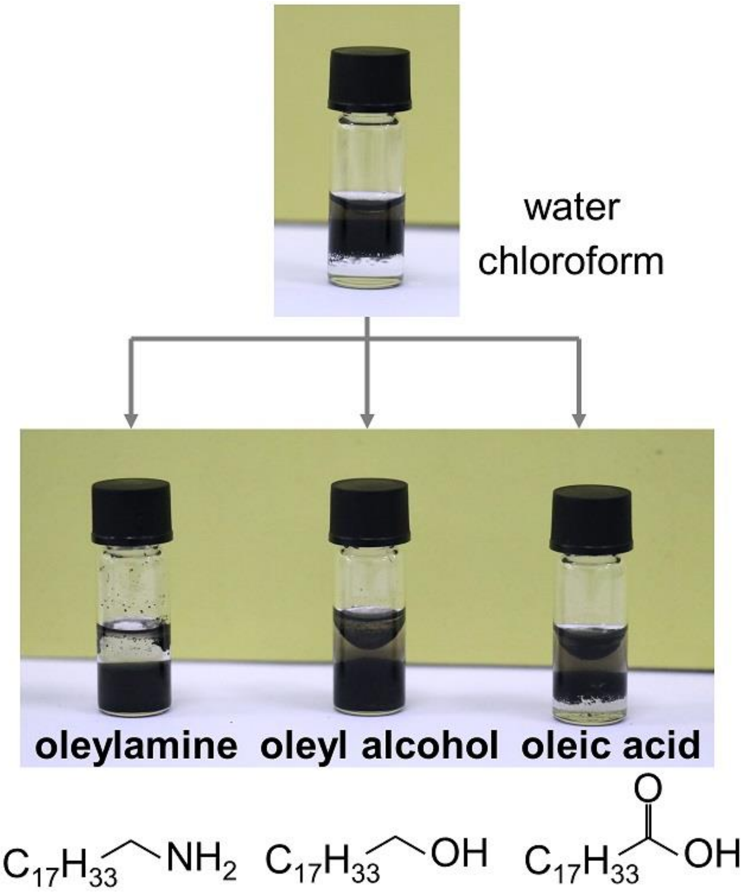

Supplementary Fig. 16 The binding affinity test of Fe_3O_4/CNT/Gent. The binding affinity test of Fe_3O_4/CNT/Gent to oleic acid, oleylamine and oleyl alcohol by observing the transfer of the Fe_3O_4/CNT/Gent from water to chloroform in the presence of these chemicals.

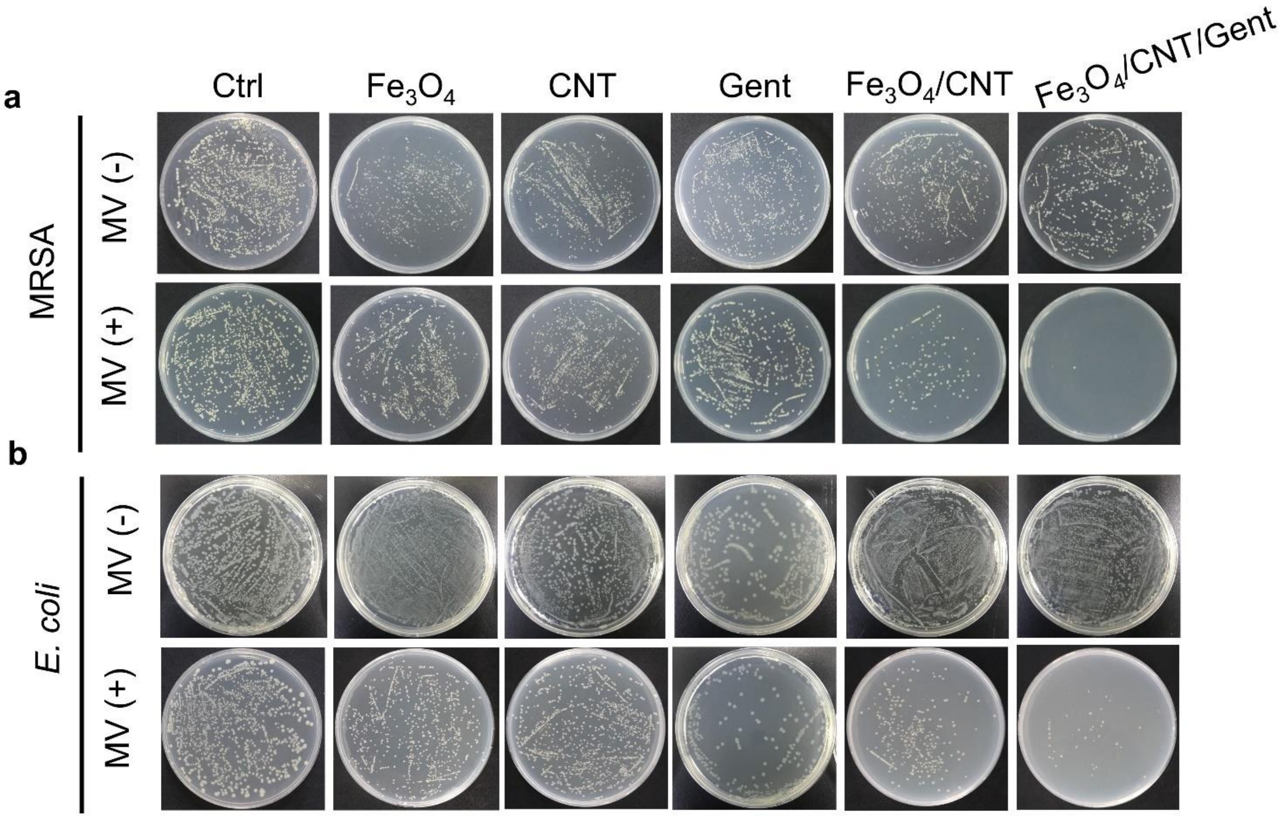

Supplementary Fig.17 In vitro antibacterial test by spread plate methods. MRSA (**a**) and *E. coli* (**b**) mixed with different samples, then exposed or unexposed to MV for 20 minutes spread onto LB agar plates and incubated at 37 °C for 20 hours.

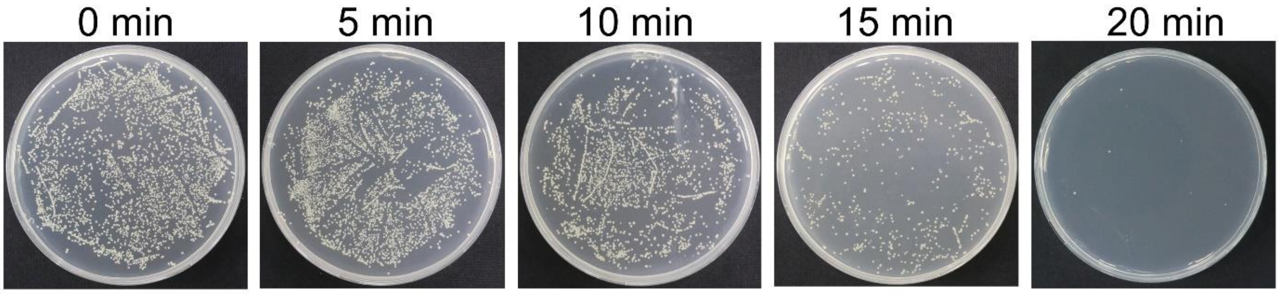

Supplementary Fig. 18 MRSA mixed with Fe_3O_4/CNT/Gent (1 mg mL^{-1}), then exposed to MV for different times (5, 10, 15 and 20 minutes) spread onto LB agar plates and incubated at 37 °C for 20 hours.

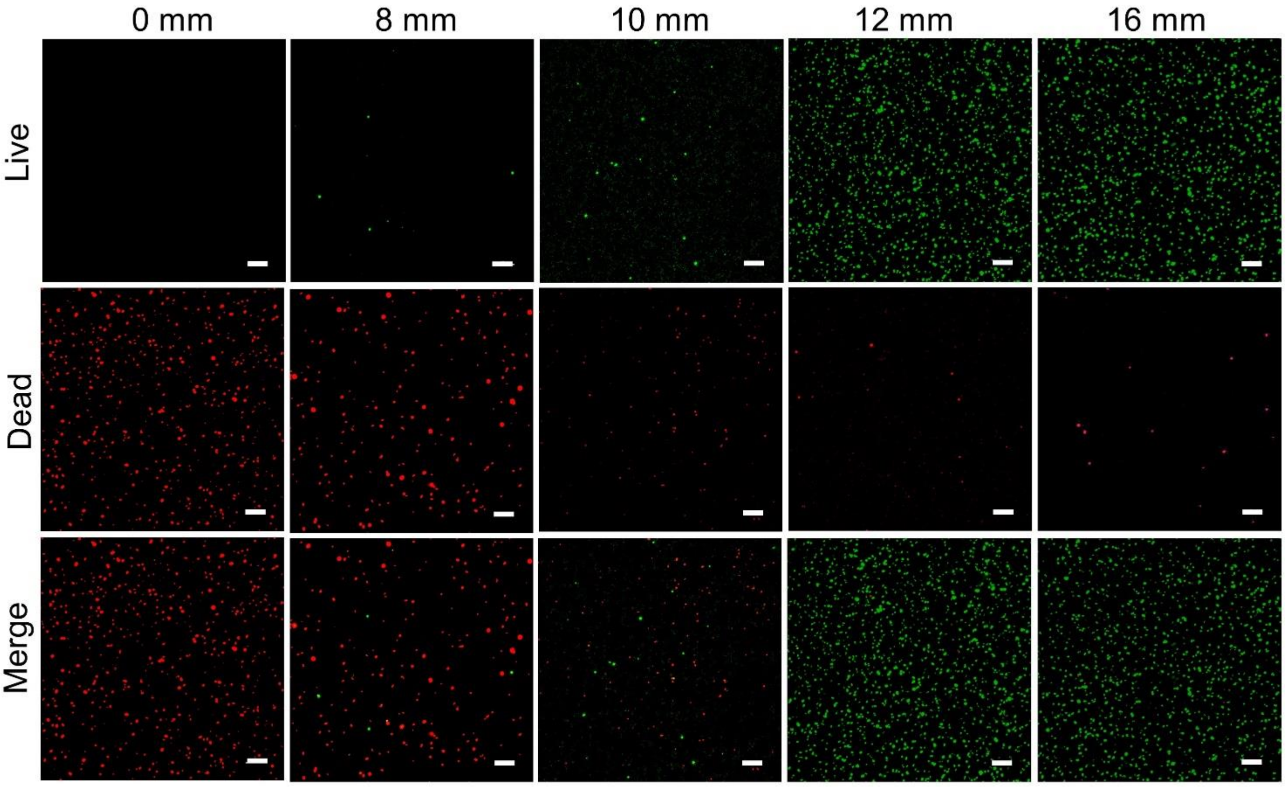

Supplementary Fig.19 Live-dead fluorescence staining of MRSA with Fe_3O_4/CNT/Gent (1 mg mL^{-1}) after MV (0.1 W cm^{-2}) excitation at different pork thickness (0, 8, 10, 12, and 16 mm). Green fluorescence was stained by SYTO9 dye which indicated live bacteria, and red fluorescence was stained by PI dye, which indicated dead bacteria. Scale bar, 10 μm.

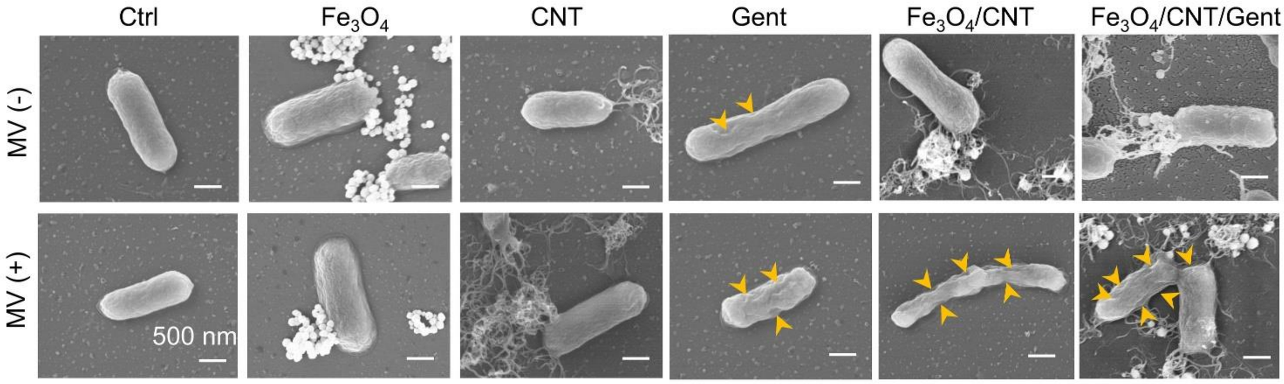

Supplementary Fig. 20 SEM images representing the morphologies and structures of *E. coli* before and after different treatments.

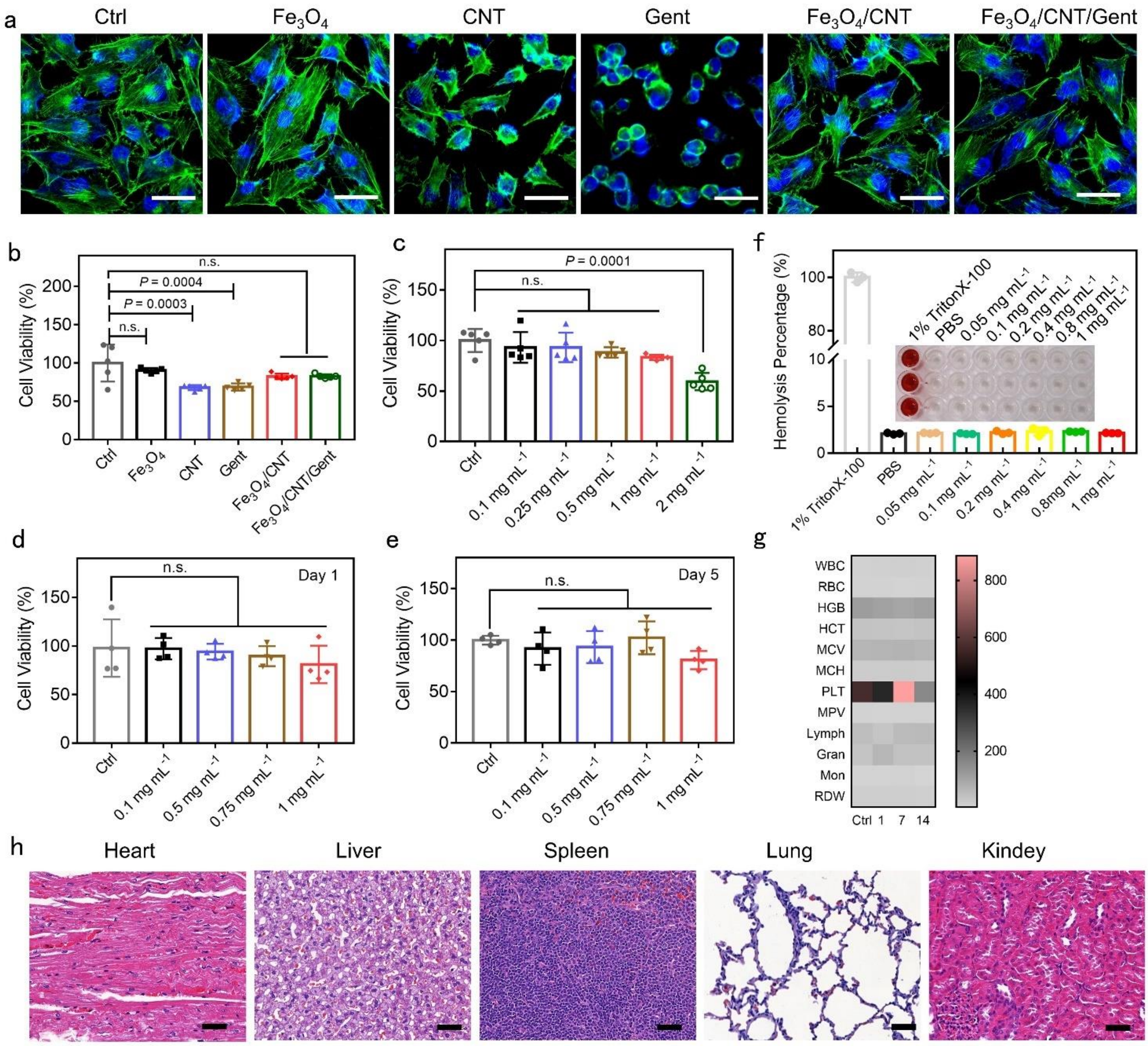

Supplementary Fig. 21 In vitro and vivo safety evaluation. **a,** Fluorescent images of MC3T3-E1 osteoblasts cocultured with different samples for one day. Scale bar, 50 μm. **b,c,** The viability of MC3T3-E1 cells cocultured with different samples (**b**) and different concentrations (**c**) of Fe_3O_4/CNT/Gent after coculturing for three days. **d,e,** The viability of NIH-3T3 cells cocultured with different concentrations of Fe_3O_4/CNT/Gent after coculturing for (**d**) one and (**e**) five days. **f,** Hemolytic efficiency of different concentrations of Fe_3O_4/CNT/Gent. Inset: The representative images demonstrating the varying degrees of hemolysis. **g,** Parameters of complete blood tests of rabbits after injection of Fe_3O_4/CNT/Gent at 1 day, 7 days, and 14 days post-injection, respectively. **h,** Histological analysis of internal organs injury in the given dose nanocapturers. Scale bar, 50 μm. Data are presented as mean ± SD from a representative experiment ($n = 5$ independent samples for **b**,**c**, $n = 3$ independent samples for **f**, $n = 4$ independent samples for **d**,**e**. The n.s. present $P > 0.05$, and P values were analysed by one-way ANOVA with

Tukey's multiple comparisons post hoc test. WBC, white blood cells; RBC, red blood cells; HGB, hemoglobin; MCH, mean erythrocyte hemoglobin; PLT, platelets; MPV, mean platelet volume; MCV, mean red blood cell volume; Gran, granulocyte; HCT, hematocrit; Mon, monocyte; RDW, red blood cell distribution width. Source data are provided as a Source Data file.

To verify the nanocapturer as a safe and effective approach for bacterial infection therapy, the cellular toxicity of nanocapturer was assessed using MC3T3-E1 osteoblasts and NIH-3T3 fibroblasts. Fluorescein isothiocyanate (FITC, green) labeled phalloidin was used to visualize the F-actin a and 4′, 6-diamidino-2-phenylindole (DAPI, blue) was used for nuclei, which can visualize initial cell adhesion and spreading activity of cells (MC3T3-E1) qualitatively. As shown in Supplementary Fig. 21a, one day after the cells separately co-cultured with physiological saline (Ctrl), Fe_3O_4, Fe_3O_4/CNT, and Fe_3O_4/CNT/Gent, the morphologies of cells, which spread well and exhibited a polygonal morphology with filopodia extensions, did not change compared with the control group. However, the MC3T3-E1 osteoblasts co-cultured with CNT, the fusiform antennae of the cells became short and shrinking. Similarly, for the Gent group, the cells withered into a ball-like structure. These results indicated that Fe_3O_4, Fe_3O_4/CNT, and Fe_3O_4/CNT/Gent were excellent cytocompatibility, but CNT and Gent exhibited cytotoxicity to some extent. The cytotoxicity of these samples was further assessed using the methyl thiazolyl tetrazolium (MTT) assay (Supplementary Fig. 21b). For MC3T3-E1, after three days of coculturing, compared with the control group, the groups of Fe_3O_4, Fe_3O_4/CNT and Fe_3O_4/CNT/Gent showed approximately 80%–90% viability, while CNT and Gent showed approximately 65% viability which was in good agreement with fluorescence staining results. Moreover, as the Fe_3O_4/CNT/Gent concentration increased, the MC3T3-E1 cells viability became lower (Supplementary Fig. 21c). Similarly, for NIH-3T3 fibroblasts, with the increase of

Fe_3O_4/CNT/Gent concentration, the cell viability did not decrease significantly after one and five days of co-cultivation, all of which were above 80% (Supplementary Fig. 21d,e). After that, the blood safety of nanocapturer was evaluated by measuring the hemolysis percentage of different concentrations of Fe_3O_4/CNT/Gent. As shown in Supplementary Fig. 21f, the hemolytic percentage of all test concentrations of Fe_3O_4/CNT/Gent in this experiment was as low as the negative control group (PBS), and far lower than that of the positive control (1% TritonX-100 is a kind of surfactant can destroy cell membrane), indicating the good blood compatibility.

To evaluate whether the nanocapturer cause tissue damage, inflammation or lesion, blood tests and histological analysis were performed. As shown in Supplementary Fig. 21g, no significant difference was observed in the blood routine (WBC, RBC, HGB, Lymph, MCH, PLT, MPV, MCV, Gran, HCT, Mon, and RDW) analysis between the control (without surgery) and Fe_3O_4/CNT/Gent groups at a given dose, indicating that nanocapturer has no appreciable toxicity and are safe for in vivo application under our experimental conditions, which was further supported by the hematoxylin and eosin (H&E) results of the internal heart, liver, spleen, lung, kidney (Supplementary Fig. 21h).

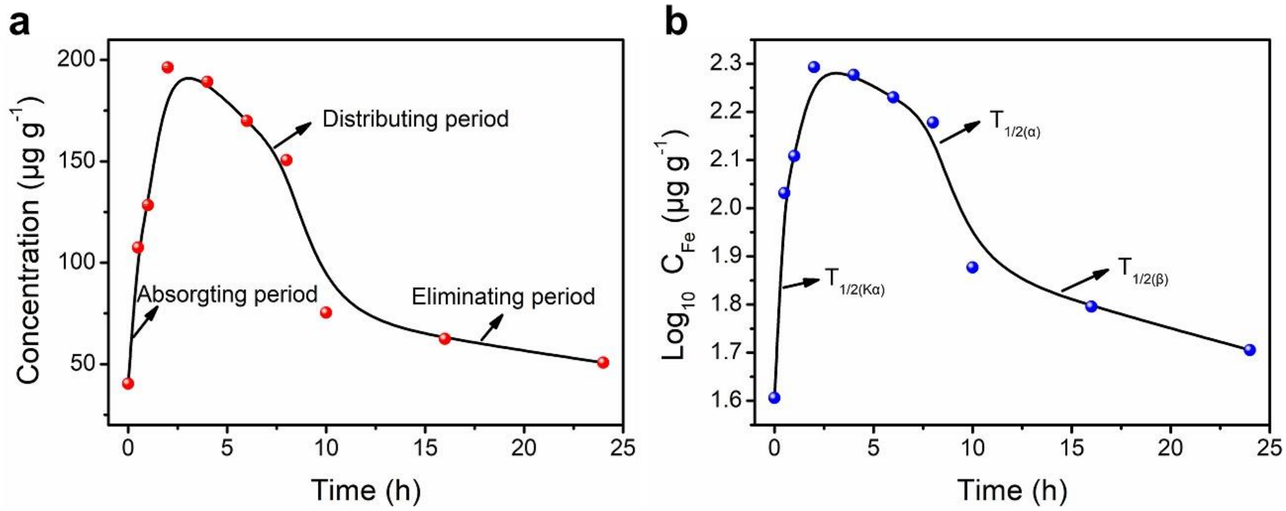

Supplementary Fig. 22 The pharmacokinetics studies of nanocapturer. **a,** The blood circulation curve of in situ injection nanocapturer. **b,** The Log_{10} C_{Fe}–T relationship curve of Fe_3O_4/CNT/Gent from the blood circulation curve. Source data are provided as a Source Data file.

The pharmacokinetics of Fe_3O_4/CNT/Gent nanocapturer was investigated. After injection the in situ, the blood circulation of Fe_3O_4/CNT/Gent nanocapturer was followed a two-compartment model (Supplementary Fig. 22a). Based on the Log_{10}(concentration)–T relationship (Supplementary Fig. 22b), we calculated that the eliminating curve constant of Fe_3O_4/CNT/Gent nanocapturer was 1.0928 μg mL^{-1} h^{-1} in the first stage and then decreased to 0.0372 μg mL^{-1} h^{-1} in the second state and 0.0122 μg mL^{-1} h^{-1} in the third state. Collectively, these data indicated that Fe_3O_4/CNT/Gent nanocapturer exhibited a superior blood circulation profile.

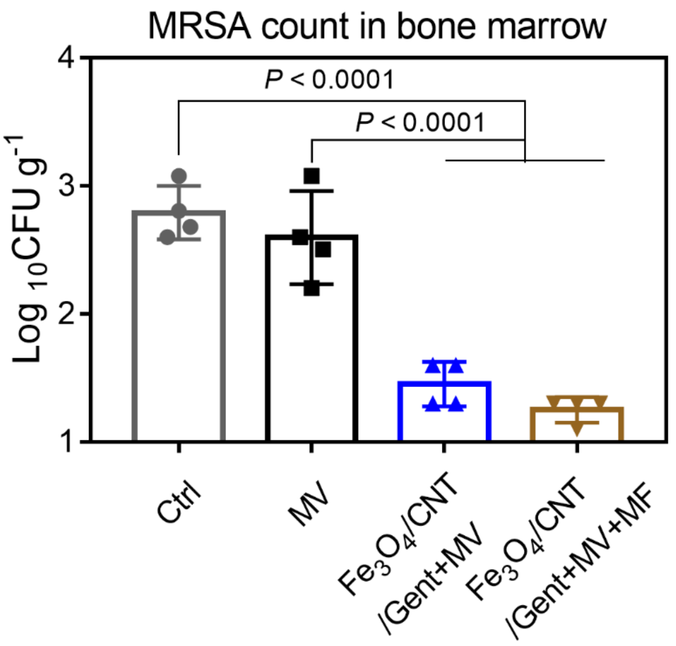

Supplementary Fig. 23 The MRSA counts in the infected bone marrow after 14 days with different treatments. Data are presented as mean ± standard deviations from a representative experiment (*n* = 4 biologically independent samples). *P* vaules were analysed by one-way ANOVA with Tukey's multiple comparisons post hoc test. Source data are provided as a Source Data file.

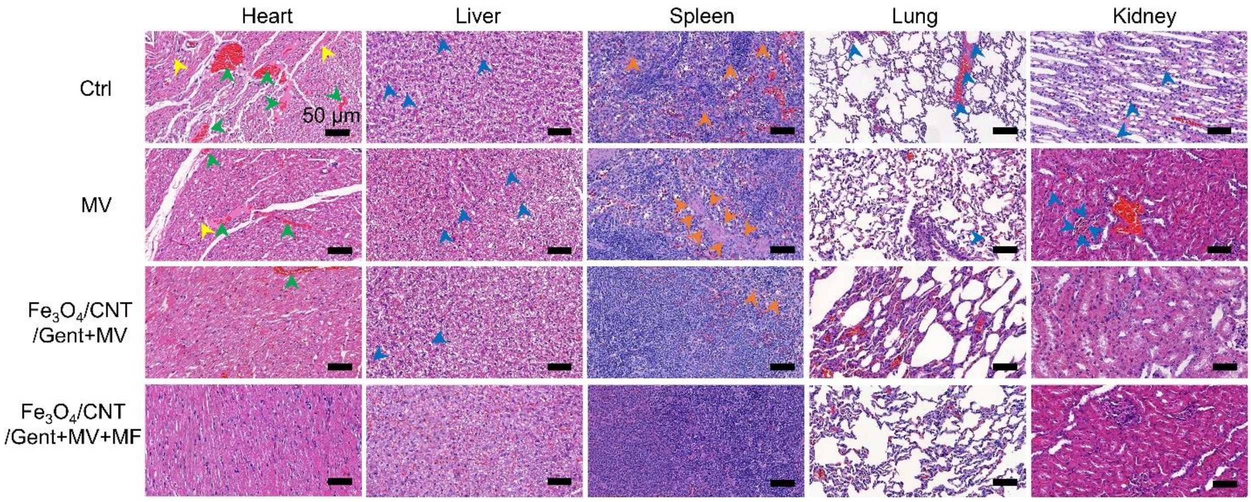

Supplementary Fig. 24 H&E staining images of heart, liver, spleen, lung and kidney after 14 days posttreatment.

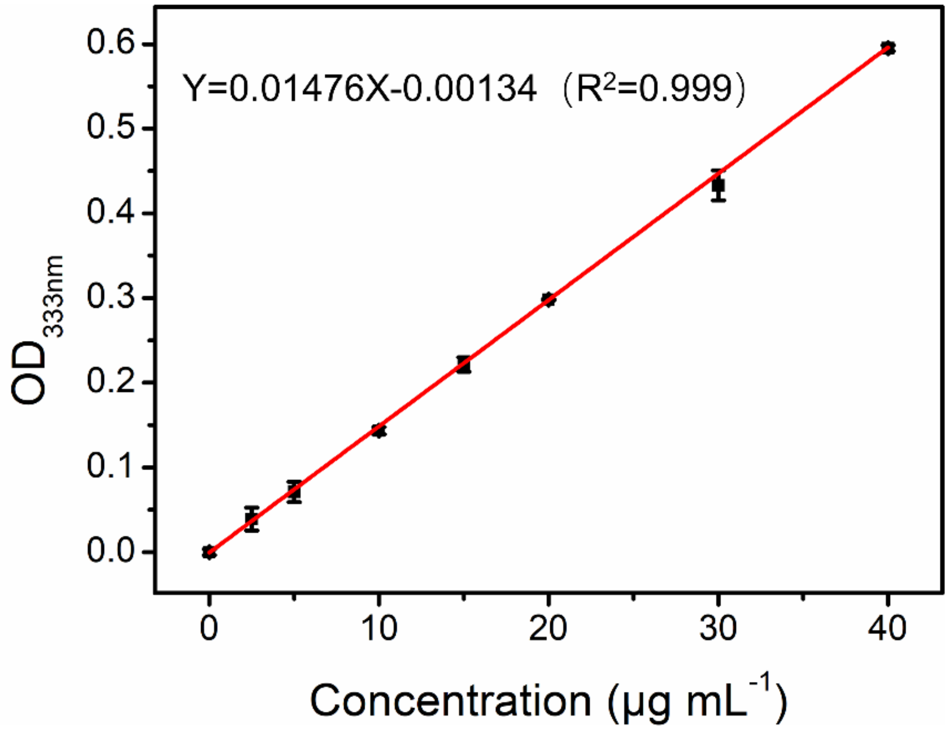

Supplementary Fig. 25 The standard curve of Gent. Data are presented as mean ± standard deviations from a representative experiment ($n = 3$ independent samples). Source data are provided as a Source Data file.

Supplementary Table 1 Parameters for calculating microwaveocalortic conversion efficiency η of Fe_3O_4/CNT

$\Sigma C_{0.9\%Nacl} \cdot m \cdot \Delta T$	ζ_s	Q_{loss}	Q_{input}	η (%)
230.81	5.73	40.32	420.18	35.7%

where m is the mass of the 0.9% NaCl solution and ΔT is the temperature increase. Q_{input} is the energy from the MW source. Q_{loss} is the heat loss to the surroundings, and η is defined as MW thermal conversion efficiency. $C_{0.9\% NaCl}$ is the specific heat capacity of the 0.9% NaCl solution.

From an energy balance of the system, the microwaveocalortic transduction efficiency can be calculated[5]. The total energy for the system is as follows:

$$Q_{input} \times \eta = Q_{heat} + Q_{loss} \tag{1}$$

Q_{input} is the energy from the MW source. Q_{heat} is the temperature of Fe_3O_4/CNT solution. Q_{loss} is the heat loss to the surroundings. η is defined as nominal microwaveocalortic conversion efficiency.

$$Q_{input} = p \times s_1 \times (t_n - t_0) \tag{2}$$

Where p is output power, s_1 is the heating surface area of the container, t_n is the highest temperature of the steady state, t_0 is the initial temperature before MW heating.

$$C_{0.9\%Nacl} = A_{water} \times C_{water} + A_{15\%Nacl} \times C_{15\%Nacl} \tag{3}$$

where A_{water} and $A_{15\% NaCl}$ are volume-weight of the water and 15% NaCl solution, respectively. C_{water} and $C_{15\% NaCl}$ are the specific heat capacity of the water and 15% NaCl solution, respectively.

$$Q_{heat} = \sum_{t=t_0}^{t=t_n} C_{0.9\%\,Nacl} \times m \times \Delta T$$

$$= C_{0.9\%\,Nacl} \times m \times (T_n - T_0) \tag{4}$$

$$Q_{loss} = \sum_{t=t'_0}^{t=t'_n} h \times s_1 \times \Delta\left(T_{Fe_3O_{4/CNT}} - T_{Env}\right) \times \Delta t$$

$$= \sum_{t=t'_0}^{t=t'_n} h \times s_1 \times \Delta(f_1(t) - f_2(t)) \times \Delta t \tag{5}$$

Where h is the heat transfer coefficient, $T_{Fe3O4/CNT}$ is the temperature of Fe_3O_4/CNT solution. T_{Env} is the ambient surrounding temperature, f_1 (t) and f_2 (t) are the function formulas of $T_{Fe3O4/CNT}$ and T_{Env} for time.

According to the definition of integral, the calculation equation can be transformed as follows:

$$Q_{loss} = \sum_{t=t'_0}^{t=t'_n} h \times s_1 \times \Delta\big(f_1(t) - f_2(t)\big) \times \Delta t$$

$$= h \times s_1 \times \sum_{t=t'_0}^{t=t'_n} [\big(f_1(t) - f_2(t)\big)]\, d_t$$

$$= h \times s_1 \times \int_{t'_0}^{t'_n} [\big(f_1(t) - f_2(t)\big)] d_t$$

$$= h \times s_1 \times \Delta S \tag{6}$$

$h \times s_1$ is determined by measuring the process of the free heat dissipation stage II after withdrawing the microwave source.

When the solution reached stable states:

$$\sum_{t=t'_0}^{t=t'_n} C_{0.9\%\,Nacl} \times m \times \frac{dT}{dt} = -h \times s_1 \times \Delta T \tag{7}$$

Supposing: $\theta = \frac{\Delta T}{T_{max}}$

$$\sum_{t=t'_n}^{t=t'_n} C_{0.9\%\,Nacl} \times m \times \frac{d\theta}{dt} = -h \times s_1 \times \Delta T$$

$$dt = -\frac{\sum_{t=t'_0}^{t=t'_n} C_{0.9\%\,Nacl} \times m}{h \times s_1} \frac{d\theta}{\theta}$$

$$\xi_s = \frac{\sum_{t=t'_0}^{t=t'_n} C_{0.9\%\,Nacl} \times m}{h \times s_1}$$

$$t = \xi_s(-ln\theta) + b \tag{8}$$

And $h \times s_1$ can be calculated by the slope of t *vs* -lnθ

The time variation value is assumed to infinite approach zero:

$lim\Delta t = \lim\,(t_n - t_{n-1}) \to 0^+$

Finally, η can be calculated as follows:

$$\eta = \frac{\sum_{t=t_0}^{t=t_n} C_{0.9\%\,Nacl} \times m \times \Delta T + Q_{loss}}{Q_{input}}$$

$$= C_{0.9\%\,Nacl} \times m \times (T_n - T_0) + \frac{\sum_{t=t_0}^{t=t_n} C_{0.9\%\,Nacl} \times m}{\xi_s} \times \int_{t_0}^{t_n} [\big(f_1(t) - f_2(t)\big)] d_t / p \times s_1(t_n - t_0) \tag{9}$$

Supplementary Table 2 Combination index of Gent combination for MCT resistant MRSA

Drug	f_a	Parameter			CI
		m	D_m	r	
Gent		0.49430	63.9838	0.98002	
MCT		1.77397	1.15001	0.99691	
Gent+MCT	99.556%				0.00729

The parameters m, D_m, and r are the slope, antilog of the x-intercept, and the linear correlation coefficient of the median-effect plot, which signifies the shape of the dose-effect curve, and the conformity of the data to the mass-action law, respectively.

Supplementary References:

1. Chen Z, et al. Soluble Ultra-Short Single-Walled Carbon Nanotubes. *J. Am. Chem. Soc.* **128**, 10568-10571 (2006).
2. Wu Q, et al. Dual-Functional Supernanoparticles with Microwave Dynamic Therapy and Microwave Thermal Therapy. *Nano Lett.* **19**, 5277-5286 (2019).
3. Kunzmann A, Andersson B, Thurnherr T, Krug H, Scheynius A, Fadeel B. Toxicology of engineered nanomaterials: Focus on biocompatibility, biodistribution and biodegradation. *Biochim. Biophys. Acta-Gen. Subj.* **1810**, 361-373 (2011).
4. Kagan VE, *et al.* Carbon nanotubes degraded by neutrophil myeloperoxidase induce less pulmonary inflammation. *Nat. Nanotechnol.* **5**, 354-359 (2010).
5. Fu C, *et al.* Microwave-Activated Mn-Doped Zirconium Metal–Organic Framework Nanocubes for Highly Effective Combination of Microwave Dynamic and Thermal Therapies Against Cancer. *ACS Nano* **12**, 2201-2210 (2018).
